# Association of antibiotics with veteran visit satisfaction and antibiotic expectations for upper respiratory tract infections

**DOI:** 10.1017/ash.2022.233

**Published:** 2022-06-23

**Authors:** Milner B. Staub, Rachael Pellegrino, Erin Gettler, Morgan C. Johnson, Christianne L. Roumie, Carlos G. Grijalva, Kaitlyn Reasoner, Robert S. Dittus, Todd Hulgan

**Affiliations:** 1 Geriatric Research, Education, and Clinical Center (GRECC), Veterans Health Administration, Tennessee Valley Healthcare System, Nashville, Tennessee; 2 Division of Infectious Diseases, Department of Medicine, Vanderbilt University Medical Center, Nashville, Tennessee; 3 Department of Medicine, Vanderbilt University Medical Center, Nashville, Tennessee; 4 Division of Pharmacoepidemiology, Department of Health Policy, Vanderbilt University Medical Center, Nashville, Tennessee (Present affiliation: Division of Infectious Diseases, Duke University School of Medicine, Durham, North Carolina [E.G.]).; 5 Infectious Diseases Section, Medical Services, Tennessee Valley Healthcare System, Nashville, Tennessee

## Abstract

**Background and objective:**

Veterans’ Affairs (VA) healthcare providers perceive that Veterans expect and base visit satisfaction on receiving antibiotics for upper respiratory tract infections (URIs). No studies have tested this hypothesis. We sought to determine whether receiving and/or expecting antibiotics were associated with Veteran satisfaction with URI visits.

**Methods:**

This cross-sectional study included Veterans evaluated for URI January 2018–December 2019 in an 18-clinic ambulatory VA primary-care system. We evaluated Veteran satisfaction via the Patient Satisfaction Questionnaire Short Form (RAND Corporation), an 18-item 5-point Likert scale survey. Additional items assessed Veteran antibiotic expectations. Antibiotic receipt was determined via medical record review. We used multivariable regression to evaluate whether antibiotic receipt and/or Veteran antibiotic expectations were associated with satisfaction. Subgroup analyses focused on Veterans who accurately remembered antibiotic prescribing during their URI visit.

**Results:**

Of 1,329 eligible Veterans, 432 (33%) participated. Antibiotic receipt was not associated with differences in mean total satisfaction (adjusted score difference, 0.6 points; 95% confidence interval [CI], −2.1 to 3.3). However, mean total satisfaction was lower for Veterans expecting an antibiotic (adjusted score difference −4.4 points; 95% CI −7.2 to −1.6). Among Veterans who accurately remembered the visit and did not receive an antibiotic, those who expected an antibiotic had lower mean satisfaction scores than those who did not (unadjusted score difference, −16.6 points; 95% CI, −24.6 to −8.6).

**Conclusions:**

Veteran expectations for antibiotics, not antibiotic receipt, are associated with changes in satisfaction with outpatient URI visits. Future research should further explore patient expectations and development of patient-centered and provider-focused interventions to change patient antibiotic expectations.

The majority of antibiotic prescribing occurs in the outpatient setting.^1^ Upper respiratory tract infections (URIs), frequently evaluated in outpatient visits, are mostly caused by viruses, rendering antibiotics unnecessary.^2^ Yet, a recent evaluation of US ambulatory-care visits demonstrated that 41% of all antibiotics were prescribed for respiratory illness and 51% of acute URI visits resulted in antibiotic prescriptions.^3^

Many providers perceive that patients desire or expect antibiotics,^4,5^ which strongly influences provider prescribing.^6–8^ Although patient expectations for antibiotics may be decreasing over time,^5,9^ a reported 50%–72% of patients expect antibiotics for URI-like symptoms.^6,10–12^ Many studies have shown increased patient satisfaction associated with antibiotic receipt;^13–15^ others showed no association.^16,17^ Studies that directly looked at patient-reported expectation for antibiotics and patient satisfaction with care found that for patients who reported expecting an antibiotic, antibiotic receipt was associated with increased satisfaction^12,18^ and not receiving an antibiotic was associated with decreased satisfaction.^10^

Only 1 study specifically included Veterans and Veterans’ Affairs (VA) facilities and demonstrated that receiving an antibiotic at an emergency department visit for URI was not associated with a difference in reported satisfaction for Veterans but was associated with increased satisfaction for non Veterans,^19^ suggesting that there are differences in what drives Veteran visit satisfaction compared to non Veterans. To our knowledge, no studies have evaluated the association of satisfaction with clinical care and antibiotic receipt for URIs in outpatient Veteran clinics. Here, we present findings from a cross-sectional study using survey data and data extracted from electronic medical record (EMR) review assessing whether receiving an antibiotic prescription during URI clinic visits was associated with Veteran satisfaction and whether Veteran self-reported expectation for antibiotics for URI was associated with Veteran satisfaction.

## Methods

### Patient population

This cross-sectional study included adult Veterans (aged ≥18 years) evaluated for URI or acute bronchitis at 18 VA Tennessee Valley Healthcare System (TVHS) outpatient clinics from January 1, 2018, through December 31, 2019. These Veterans were previously identified as part of a quality improvement project at VA TVHS.^20^ From March to May 2020, eligible Veterans were screened. Veterans who had died since their URI visit, had documented dementia, or had not been evaluated in-person were excluded. Veterans who asked to be removed from the study after receiving a survey were also excluded.

### Outcomes

The study’s primary outcome was total satisfaction with URI visit measured by the Patient Satisfaction Questionnaire Short-Form (PSQ-18) (RAND Corporation, Santa Monica, CA).^21^ The PSQ-18 is a validated 18-item survey that assesses patient satisfaction with clinical care using a 5-point Likert scale (ie, strongly disagree, disagree, uncertain, agree, strongly agree) and yields a total score of 18–90 points, with higher scores indicating higher overall satisfaction.^21^ Secondary outcomes included satisfaction by PSQ-18 subscales. The survey addressed 7 subscales of satisfaction: general satisfaction, technical quality, interpersonal manner (of the provider), communication, financial aspects, time spent with the doctor, and accessibility and convenience. The PSQ-18 has been used in >50 published studies, often to evaluate different aspects of outpatient clinical care.

We also evaluated the association of Veteran expectation for antibiotics for URIs with URI clinic visit satisfaction in all participants and separately in the subgroup of Veterans who accurately remembered the URI clinical visit.

### Survey development

The study survey included the PSQ-18 and additional items assessing patient expectations for antibiotics for URIs, perception of how antibiotics affected their URI clinical visit satisfaction, whether the Veteran remembered the specific URI clinical visit, and whether the Veteran remembered whether an antibiotic was prescribed. The survey also included a prompt informing the Veteran that he or she had been evaluated in a VA primary care clinic and diagnosed with URI or acute bronchitis between January 2018 and December 2019 and listed examples of URI-related symptoms for which they may have been evaluated. The Veteran was then asked to think about that visit while answering the survey (Supplementary Table 1).

Extensive evaluations and refinements of the study survey were conducted prior to distribution. In 3 rounds of cognitive testing, VA TVHS Veterans who were not study eligible were asked to complete the survey and to provide feedback on whether the prompt and survey items were easily understood and how to improve the survey.

### Survey dissemination

A unique code was assigned to all eligible Veterans and included on the mailed survey to reduce risk of unintended disclosure and to link EMR review information with survey data. Surveys were mailed beginning July 1, 2020. The survey was mailed 2 additional times to nonresponders to increase participation. All information was deidentified and entered into the Veterans’ Affairs REDCap database (Vanderbilt University, Nashville, TN). This study was approved by the VA TVHS Institutional Review Board.

### Survey scoring

According to PSQ-18 scoring instructions, skipped items were excluded^22^ and all scores were adjusted to the scale of 90. We included all surveys with at least 1 item answered. Satisfaction subscales consisted of either 2 or 4 items per scale. All skipped or missed items for each subscale were excluded, and an average score was calculated, ranging from 1 to 5. We excluded Veterans who skipped all items in a particular subscale from that subscale analysis.

### Study exposure

EMR reviews were conducted separately to ascertain patient and visit-specific data, including whether the patient was prescribed an antibiotic. The review team comprised 4 independent EMR reviewers: 1 who reviewed EMRs throughout the project, 1 who performed EMR reviews only initially, and 2 who performed EMR reviews later in the project. All reviewers were aware of the study hypothesis at the time of EMR extraction. Percentage agreement was calculated among concurrent reviewers at 2 separate times due to the change in reviewers.

### Study covariates

Covariates that could affect prescribing of an antibiotic for URI and/or the Veteran’s satisfaction with clinical care were assessed and included in adjusted models. We collected data on Veteran sex, age, and home-county 2013 National Center for Health Statistics (NCHS) urban–rural classification, based on the Office of Management and Budget’s metropolitan statistical areas.^23^ We collected the following patient clinical data: status for smoking, congestive heart failure (CHF), pulmonary disease, and immunosuppression, estimated glomerular filtration rate (eGFR), and documented insurance status. We also collected the following visit and provider covariate data: season of clinic visit, clinic site, provider, provider type, whether medications for URI symptoms were prescribed, and whether the patient revisited or communicated with the clinic for the same complaint within 1 week (Supplementary Table 2).

### Analysis

#### Statistical analysis

We performed a *t* test for continuous and binominal variables and a 2-sample proportion test for categorical variables to assess whether the difference in means between those that did and those that did not receive an antibiotic was different from zero, and we computed 95% confidence intervals (95% CI) around the estimated difference.

For the primary analysis of satisfaction associated with antibiotic receipt and for the secondary analysis of satisfaction associated with Veteran expectation for an antibiotic, we used multivariate multiple imputation^24^ to derive values for missing eGFR. We generated 10 data sets using sex, age, NCHS urban–rural county classification, smoking status, chronic pulmonary disease status, CHF status, immunosuppressed status, documented insurance status, days from visit to survey response, receipt of a nonantibiotic prescription for URI symptoms, and return visit or call within 1 week for imputation.

We applied multivariable linear regression to assess difference in total satisfaction associated with receiving compared to not receiving an antibiotic, adjusted for confounding patient and clinic characteristics listed above in addition to eGFR, provider type, and Veteran self-reported expectation, clustered by clinic site.

To evaluate differences in mean satisfaction subscales among those who received an antibiotic compared to those who did not, we used the Wilcoxon rank-sum test. We used multivariable linear regression to assess the association of Veteran expectations for antibiotics for URI on total visit satisfaction.

#### Secondary analysis to address potential effect modification

Prior studies evaluating patient satisfaction with clinical visits found that older age groups had increased satisfaction.^14,25^ Therefore, to assess for potential effect modification, we evaluated satisfaction using age groups, stratified by quartiles, substituted for age in the multivariable linear regression.

#### Secondary and subanalyses to address potential biases

We mailed surveys from July to September 2020, but visits occurred as early as January 1, 2018. To address potential recall bias, we calculated time from clinic visit to survey response for each Veteran. We chose single dates in July, August, and September 2020 (coinciding with mailing dates) as standard response dates, and we calculated the days from each Veteran’s clinic visit appointment to survey response. We evaluated difference in days from initial visit to survey response among those who received an antibiotic compared to those who did not. We also assessed difference in satisfaction based on time from visit to survey response.

To further address potential recall bias, we included 2 items to assess the Veteran’s memory of the visit (Supplementary Table 1). For Veterans who remembered the visit or were unsure of remembering the visit but definitely remembered receiving or not receiving an antibiotic, we compared reported antibiotic receipt to actual antibiotic receipt determined by EMR review. We calculated the positive predictive value for remembering receiving an antibiotic and the negative predictive value for remembering not receiving an antibiotic. We evaluated difference in satisfaction associated with Veterans accurately remembering the visit compared to other responders.

We performed additional subgroup analyses among those who accurately remembered the visit. We assessed difference in satisfaction and in time from visit to survey response among those who received an antibiotic compared to those who did not. We also assessed difference in satisfaction based on Veteran expectation for antibiotics and antibiotic receipt.

For subgroup analyses of Veterans who accurately remembered the visit, we used multivariable linear regression adjusted for sex and age to assess difference in satisfaction and in days from time of visit to survey response among those who received and did not receive an antibiotic. We used simple linear regression, due to smaller sample size, to assess difference in satisfaction based on antibiotic expectation and antibiotic receipt.

Acknowledging that surveys often have lower than desired response rates, we also evaluated whether responders were representative of the entire identified patient population by comparing the distribution of Veteran and visit characteristics and the percentage of Veterans who received antibiotics between responders and nonresponders.

## Results

### Survey responses

**Fig. 1.**
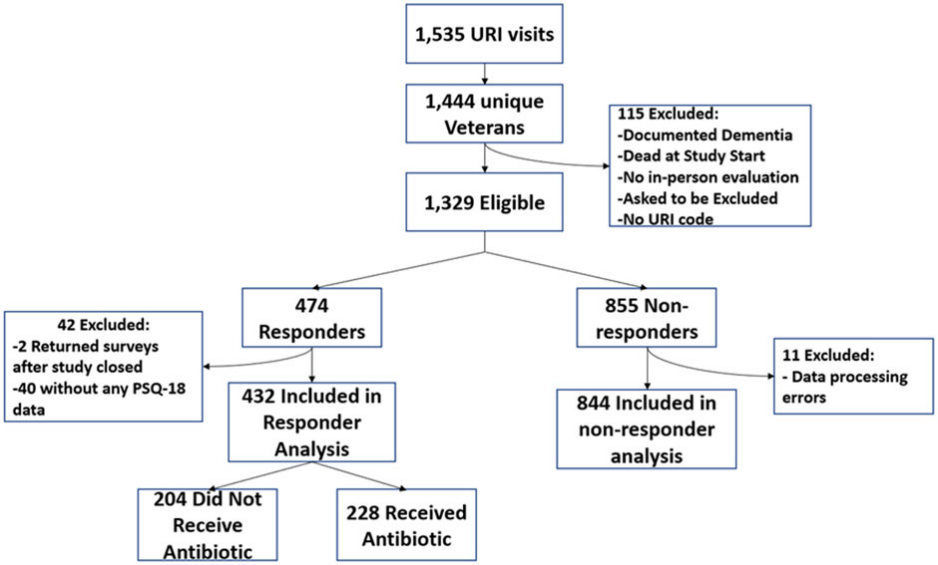
Disposition and responses of Veterans with upper respiratory infection visits.

We identified 1,535 URI visits from 1,444 unique Veterans, of whom 1,329 were eligible and 474 responded; 42 surveys were excluded (2 were returned after the study closed and 40 were returned blank). The final analysis included 432 Veteran responses, for a 33% response rate (Fig. 1). Respondents had a mean age of 64.0 (±11.9) years and 62 (14.4%) were female. For antibiotic receipt, percentage agreement among initial EMR reviewers was 92% and among later EMR reviewers was 100%. We did not report kappa estimates because the equation could not be used due to 100% agreement.

Among 228 Veterans (52.8%) who received an antibiotic compared to 204 (47.2%) who did not, there was no significant difference in sex, age, urban–rural classification based on home address, percentage who smoked, had underlying pulmonary disease, CHF, or immunosuppression, mean eGFR, or percentage with documented insurance. For Veterans who received an antibiotic compared with those who did not, the percentage of nurse practitioner visits was significantly higher (difference, 20.7%; 95% CI, 12.0%–29.4%; *P* < .001); however, the percentage of physician visits was significantly lower (difference, −15.9%; 95% CI, −25.1% to −6.8%; *P* = .001). For Veterans who received an antibiotic compared with those who did not, the percentage who received supportive medications to address URI symptoms was higher (difference, 14.4%; 95% CI, 5.1%–23.8%; *P* = .003), the percentage who called or revisited within 1 week was higher (difference, 8.4%; 95% CI, 3.4%–13.5%; *P* = .001), and the mean days from clinic visit to survey response was higher (120.0 days; 95% CI, 82.1–157.9; *P* < .001) (Table 1).

**Table 1.** Veteran and Visit Characteristics by Antibiotic Receipt

Veteran and Visit Characteristics	Did Not Receive Antibiotic (N = 204), No. (%)	Received Antibiotic (N = 228), No. (%)	Difference (95% CI)	*P* Value^a^
Sex, female	29 (14.2%)	33 (14.5%)	0.3% (−6.4% to 6.9%)	.939
Age, y^b^	63.7 (±12.3)	64.2 (±11.6)	0.5 (−1.8 to 2.8)	.668
**Urban–rural county classification by Veteran address**				
Large central metro	21 (10.3%)	19 (8.3%)	−2.0% (−7.5% to 3.5%)	.483
Large fringe metro	41 (20.1%)	52 (22.8%)	2.7% (−5.0% to 10.4%)	.494
Medium metro	59 (28.9%)	67 (29.4%)	0.5% (−8.1%, 9.0%)	.916
Small metro	19 (9.3%)	12 (5.3%)	−4.1% (−9.0%, 0.9%)	.103
Micropolitan	44 (21.6%)	55 (24.1%)	2.6% (−5.4% to 10.5%)	.528
Non-core	20 (9.8%)	23 (10.1%)	0.3% (−5.4% to 5.9%)	.922
**Visit season**				
Winter (Dec–Feb)	97 (47.6%)	125 (54.8%)	7.3% (−2.1% to 16.7%)	.131
Spring (Mar–May)	46 (22.6%)	29 (12.7%)	−9.8% (−17.0%, −2.6%)	.007
Summer (Jun–Aug)	17 (8.3%)	37 (16.2%)	7.9% (1.8% to 14.0%)	.013
Fall (Sep–Nov)	44 (21.6%)	37 (16.2%)	−5.3% (−12.7% to 2.1%)	.156
**Provider type**				
Physician	139 (68.1%)	119 (52.2%)	−15.9% (−25.1% to −6.8%)	.001
Nurse practitioner	49 (24.0%)	102 (44.7%)	20.7% (12.0% to 29.4%)	<.001
Physician assistant	16 (7.8%)	6 (2.6%)	−5.2% (−9.4% to −1.0%)	.014
Unknown	0 (0.0%)	1 (0.4%)	0.4% (−0.4% to 1.3%)	.344
Received nonantibiotic prescription for URI symptoms	94 (46.1%)	138 (60.5%)	14.4% (5.1% to 23.8%)	.003
Called or seen within 1 week of URI visit	7 (3.4%)	27 (11.8%)	8.4% (3.5% to 13.3%)	.001
Smoker^c^	42 (20.6%)	56 (24.6%)	4.0% (−3.9% to 11.9%)	.324
Underlying pulmonary disease^d^	8 (3.9%)	18 (7.9%)	4.0% (−0.4% to 8.4%)	.078
Underlying congestive heart failure	16 (7.8%)	19 (8.3%)	0.5% (−4.7% to 5.7%)	.852
Estimated glomerular filtration rate (mg/dL)^b^	77.5 (±20.5)	77.5 (±22.9)	−0.0 (−4.4 to 4.4)	.996
Immunosuppressed	4 (2.0%)	2 (0.9%)	−1.1% (−3.4% to 1.2%)	.348
Insurance documented	164 (80.4%)	176 (77.2%)	−3.2% (−10.9% to 4.5%)	.417
Days since visit, d^b^,^e^	593.0 (±239.1)	713.0 (±156.9)	120.0 (81.2 to 158.7)	<.001

a Mean difference and 95% confidence intervals calculated using 2-sample *t* test for continuous and binomial data and using two-sample proportion test for categorical data. *P* values reported as statistical significance assessing whether difference in means was different from 0.

b Variables are reported as mean ± standard deviation in units specified.

c Cigarettes, cigars, or illicit substances smoked within 1 year prior to visit.

d Excludes obstructive sleep apnea and asthma.

e Calculated from set dates depending on the survey round to which the Veteran responded.

### Primary outcomes

We detected no difference in mean total satisfaction for Veterans who received an antibiotic (67.7 points) compared to those who did not (66.9 points) (adjusted score difference, 0.6 points; 95% CI, −2.1 to 3.3). Additionally, there was no significant difference in any satisfaction subscale scores between those who received and those who did not receive antibiotics (Table 2). There was no statistically significant difference in total satisfaction associated with any measured covariates except for Veteran expectation for antibiotics (Table 3). For Veterans who expected an antibiotic compared to those who did not, the mean total satisfaction score was 4.4 points lower (95% CI, −7.3 to −1.6 points; *P* = .005) (Table 3).

**Table 2.** Subscale Satisfaction Mean Scores by Antibiotic Receipt

Satisfaction Subscale Score	No Antibiotic (N=199–203)^a^	Antibiotic (N=224–228)^a^	*P* Value^b^
General satisfaction	3.9 (±0.9)	3.9 (±0.9)	.752
Technical quality	3.4 (±0.5)	3.5 (±0.5)	.537
Interpersonal manner	4.2 (±0.7)	4.2 (±0.8)	.505
Communication	4.2 (±0.8)	4.2 (±0.8)	.671
Financial	4.2 (±0.7)	4.3 (±0.8)	.295
Time spent with doctor	3.0 (±0.4)	3.0 (±0.5)	.773
Accessibility & convenience	3.6 (±0.9)	3.7 (±0.8)	.395

a If Veterans answered at least one item within a subscale, they were included; however, some Veterans did not answer any items within a specific subscale and therefore the total N used in calculating means scores differed. The range of responders for all subscales is presented here.

Subscale scores reported as mean score from 1–5 (± standard deviation).

b *P* values generated using Wilcoxon rank-sum analysis for nonparametric data and assessed whether the means differed significantly from one another.

**Table 3.** Change in Total Satisfaction Score Based on Veteran and Visit Characteristics

Characteristic	Change in Satisfaction Score, Points	95% CI	*P* Value^a^
Received antibiotic	0.6	−2.1 to 3.3	.648
Sex, female	0.9	−3.1 to 4.8	.651
Age (change per year increase)	0.1	−0.0 to 0.2	.189
**Urban–rural county classification**			
Large central metro	Reference		
Large fringe metro	0.1	−3.4 to 3.6	.932
Medium metro	−1.1	−5.6 to 3.3	.590
Small metro	−2.5	−7.6 to 2.6	.317
Micropolitan	−2.0	−5.1 to 1.0	.176
Non-core	−3.2	−8.0 to 1.6	.179
**Visit season**			
Winter (Dec–Feb)	Reference		
Spring (Mar–May)	1.5	−0.6 to 3.6	.156
Summer (Jun–Aug)	0.5	−2.1 to 3.0	.709
Fall (Sep–Nov)	−0.1	−3.2 to 2.9	.917
**Provider type**			
Physician	Reference		
Nurse practitioner	2.3	−1.0 to 5.6	.161
Physician assistant	−0.6	−7.2 to 5.9	.837
Received medication for URI symptoms	−1.4	−3.6 to 0.8	.202
Called or seen within 1 week	−3.5	−7.3 to 0.3	.069
Smoker ^b^	−0.1	−2.7 to 2.6	.961
Underlying pulmonary disease^c^	−1.9	−5.3 to 1.5	.254
Underlying congestive heart failure	0.6	−2.5 to 3.7	.684
eGFR (per 1 mg/dL increase)	−0.0	−0.1 to 0.0	.086
Immunosuppressed	3.1	−0.9 to 7.1	.123
Insurance documented	2.0	−1.1 to 5.1	.181
Days since visit (change per 1 day increase)	0.0	−0.0 to 0.0	.200
Expected antibiotics	−4.4	−7.3 to −1.6	.005

Note. CI, confidence interval; eGFR, estimated glomerular filtration rate.

a Mean difference and 95% confidence intervals calculated using 2-sample *t* test for continuous and binomial data and using two-sample proportion test for categorical data. *P* values reported as statistical significance assessing whether difference in means different from 0.

b Cigarettes, cigars, or illicit substances smoked within 1 year prior to visit.

c Excludes obstructive sleep apnea and asthma.

### Secondary outcomes

After stratifying by age quartiles and adjusting for confounders, the mean total satisfaction score for Veterans aged 57–66 years was higher than for Veterans aged <57 years (adjusted difference, 3.2 points; 95% CI, 0.7–5.6; *P* = .014). For Veterans aged 67–72 years and Veterans aged >72 years, compared to Veterans aged <57 years, there was no significant difference in mean total satisfaction score.

### Difference in satisfaction and days from visit to survey response by accuracy of veteran memory

Of the 432 Veterans who responded to the survey, 215 (49.8%) reported that they remembered or possibly remembered the visit and reported that they remembered receiving or not receiving an antibiotic. The positive predictive value of the Veteran reporting that they remembered receiving an antibiotic was 95 (64.2%) of 148. The negative predictive value of the Veteran reporting that they did not receive an antibiotic was 32 (47.8%) of 67. The 127 Veterans who accurately remembered the visit (95 received an antibiotic and 32 did not) were included in subgroup analyses.

### Subgroup analysis outcomes

For 127 Veterans (29.4%) who accurately remembered compared with all other 305 responders (70.6%), there was no difference in days from clinic visit to survey response (adjusted difference, 25.9 days; 95% CI, −17.3 to 69.2; *P* = .239) or mean satisfaction (adjusted score difference, −0.3 points; 95% CI, −1.7 to 2.2; *P* = .801).

Among those who accurately remembered, for those 95 (74.8%) who received an antibiotic compared with those 32 (25.2%) who did not, there was no difference in total satisfaction score (adjusted difference, 1.0 points; 95% CI, −2.8 to 4.8; *P* = .602). However, in Veterans who received an antibiotic, there was a significant increase in days from time of visit to survey response after adjusting for sex and age (adjusted days, 167.1; 95% CI, 95.7–238.5; *P* < .001).

**Fig. 2.**
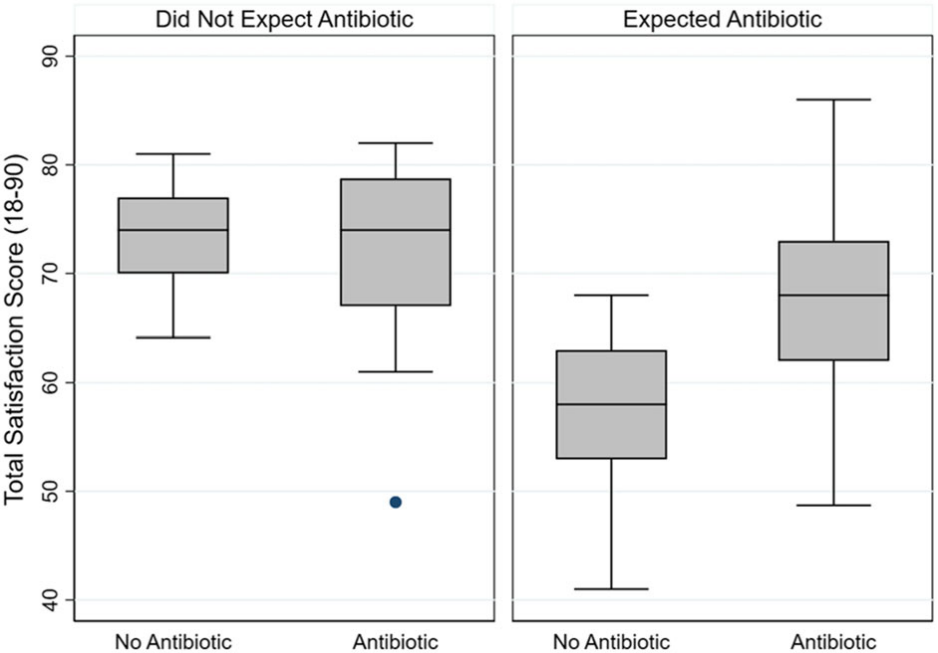
Total satisfaction by Veteran expectation for antibiotic and antibiotic receipt for Veterans who accurately remembered the visit (n = 127) and whether an antibiotic was received or not. The box plot graphs show the median and interquartile satisfaction scores (range, 18–90) among Veterans who were determined to have accurately remembered their clinical visit for upper respiratory tract infection (URI). Satisfaction scores for Veterans who reported that they did not expect an antibiotic are shown in the left box categorized into Veterans who did not receive an antibiotic and Veterans who did receive an antibiotic. These scores are compared to satisfaction scores for Veterans who did report expecting an antibiotic shown in the right box and are also categorized into Veterans who did not receive an antibiotic and Veterans who did receive an antibiotic. Veterans who expected an antibiotic regardless of antibiotic receipt had lower satisfaction scores and Veterans who expected an antibiotic but did not receive one had much lower scores.

For the 127 Veterans who accurately remembered the visit, 85 reported agreeing or disagreeing with expecting an antibiotic for URI visits. Compared with Veterans who reported not expecting an antibiotic and who did not receive an antibiotic, Veterans who reported expecting an antibiotic but did not receive one had a significantly lower unadjusted mean total satisfaction score (difference, −16.6 points; 95% CI, −24.6 to −8.6; *P* < .001) (Fig. 2).

### Nonresponder analysis outcomes

Overall, 844 Veterans who did not respond to the survey were included in the nonresponder analysis, after excluding 11 Veterans due to data-processing errors. Compared to responders, there was no difference in percentage who received an antibiotic (difference, −1.4%; 95% CI, −7.2% to 4.4%; *P* = .647). The nonresponder group was younger, more often female, had fewer calls or return visits within 1 week, smoked more, had less CHF, significantly higher eGFR and fewer with documented insurance (Supplementary Table 3).

## Discussion

Unnecessary antibiotic prescribing for URIs is high in the Veteran population.^26^ VA clinical providers prescribe antibiotics for URIs in part because they perceive that Veterans expect antibiotics for URIs and that antibiotic prescribing affects patient satisfaction.^27^ Here, we have demonstrated that in a large, VA ambulatory clinic system, receiving an antibiotic prescription for URI was not associated with patient satisfaction unless the Veteran expected an antibiotic. For Veterans who expected an antibiotic but did not receive one, mean total satisfaction with the visit was 22% lower.

Data exploring the association between antibiotic receipt and patient satisfaction for Veterans evaluated for URI are scarce.^19^ Although prior studies have assessed the effect of patient expectations for antibiotics on reported patient satisfaction for URI visits and showed similar findings,^10,12,18^ none evaluated the Veteran population. The decision to prescribe antibiotics is complex and influenced by many clinical and nonclinical factors.^8,26–28^ Nevertheless, our findings suggest that working with patients to understand what drives expectations could inform patient-focused interventions to reduce URI prescribing. Previous research has shown that it is feasible to change patients’ expectations for antibiotics for URIs.^29^

Additionally, communicating these findings to providers may empower them to assess and address patients’ expectations by implementing successful interventions like communications training.^30–32^ Patient satisfaction with URI visits has been shown to be associated with receiving information or reassurance from their provider, especially for patients who did not receive antibiotics,^12^ underscoring the importance of providing resources and tools to help providers improve patient engagement, to address patients’ expectations, and to reduce unnecessary antibiotic prescribing.

This study had several limitations. Low response is a potential limitation; however, 33% is consistent with previously reported response rates^13^ and nonresponder and responder antibiotic prescription rates were similar. Additionally, although there were differences in responder and nonresponder characteristics, none of the characteristics in the responders, aside from documented expectation, were significantly associated with a difference in mean total satisfaction. There was substantial time between visits and survey response for most responders, but in analyses evaluating recall bias, time from visit to survey response did not affect visit satisfaction scores or accuracy of the Veteran’s memory. Planned subgroup analyses with Veterans who accurately remembered the visit were similar to the larger responder population. This study was conducted in a single VA system; however, the system includes 18 clinics across Tennessee and Kentucky in rural and urban settings and includes both VA clinics and contract clinics, which provide care for Veterans but are run similarly to private practice clinics. Finally, these findings may not be directly generalizable to other populations outside the VA system; however, our findings corroborate previous findings in non Veterans, indicating that these findings are likely relevant to a wider outpatient population.

In conclusion, antibiotic receipt alone does not determine Veteran satisfaction with URI visits. Patient expectations of receiving or not receiving an antibiotic were an important factor that significantly affected satisfaction scores. Further studies evaluating how best to assess, understand, and address Veteran expectations for antibiotics for URIs are needed.

## Supplementary material

For supplementary material accompanying this paper visit https://doi.org/10.1017/ash.2022.233. click here to view supplementary material
