# Supplementary material for: Association of antibiotics with veteran visit satisfaction and antibiotic expectations for upper respiratory tract infections
Source: Antimicrob Steward Healthc Epidemiol. 2022 Jun 23;2(1):e100. doi: 10.1017/ash.2022.233 (PMC9726549; doi:10.1017/ash.2022.233)
Supplement: Supplementary file 1 [file S2732494X22002339sup001.docx]

**Supplemental Table 1**: **Items and Responses Included in Mailed Survey**

| Item | Strongly Agree | Agree | Uncertain | Disagree | Strongly Disagree |
| --- | --- | --- | --- | --- | --- |
| 1. My doctor was good about explaining the reason for medical tests if they were ordered. |  |  |  |  |  |
| 1. I think my doctor’s office had everything needed to provide medical care. |  |  |  |  |  |
| 1. The medical care I received was just about perfect. |  |  |  |  |  |
| 1. My doctor made me wonder if the diagnosis was correct. |  |  |  |  |  |
| 1. I felt confident that I could get the medical care I needed without being set back financially. |  |  |  |  |  |
| 1. When I went for these symptoms, they were careful to check everything when treating and examining me. |  |  |  |  |  |
| 1. I had to pay for more of my medical care than I can afford. |  |  |  |  |  |
| 1. I had easy access to the medical specialists I needed. |  |  |  |  |  |
| 1. Where I got medical care, people have to wait too long for emergency treatment. |  |  |  |  |  |
| 1. My doctor acted too businesslike and impersonal towards me. |  |  |  |  |  |
| 1. My doctor treated me in a very friendly and courteous manner. |  |  |  |  |  |
| 1. My doctor hurried too much when they were treating me. |  |  |  |  |  |
| 1. My doctor ignored what I told them. |  |  |  |  |  |
| 1. I had some doubts about the ability of the doctor who treated me. |  |  |  |  |  |
| 1. My doctor spent plenty of time with me. |  |  |  |  |  |
| 1. I found it hard to get an appointment for medical care right away. |  |  |  |  |  |
| 1. I was dissatisfied with some things about the medical care I received. |  |  |  |  |  |
| 1. I was able to get medical care when I needed it. |  |  |  |  |  |
| 1. When I visit my doctor with these symptoms, I hope she he/she will give me an antibiotic prescription. |  |  |  |  |  |
| 1. I expect an antibiotic when I see my doctor for these symptoms. |  |  |  |  |  |
| 1. My satisfaction with my clinic visit depended on whether I received an antibiotic or not. |  |  |  |  |  |
| 1. I was able to remember the specific visit in 2018 or 2019 when I presented with the symptoms described above. |  |  |  |  |  |
| 1. I remember receiving an antibiotic during this specific visit for my cold-like symptoms. |  |  |  |  |  |

**Supplemental Table 2:** **Definitions for Veteran and Visit Characteristics Extracted from the EMR Identified as Potential Confounders**

| **Characteristic** | **Definition** |
| --- | --- |
| **Veteran Age** | Age in years as listed in EMR at time of visit. If Veteran is older than age 89, listed as 89. |
| **Gender** | As listed in EMR at time of visit |
| **Domicile Rurality** | 2013 National Center for Health Statistics (NCHS) urban–rural classification:   1. large, central metropolitan counties (population>1 million with at least 250,000 residents of principal city) 2. large, fringe metropolitan counties (>1 million population and not classified as NCHS-1) 3. medium metropolitan counties (population 250,000–999,999) 4. small metropolitan counties (population<250,000) 5. micropolitan areas (population 10,000–50,000) 6. noncore counties (outside all other areas) |
| **Season of Clinic Visit** | Winter: December, January, February; Spring: March, April, May; Summer: June, July, August; Fall: September, October, November |
| **Specific Clinic** | Listed as one of the 18 possible VA community-based outpatient clinics in which patient was evaluated |
| **Provider Type** | Defined as physician (MD/DO), nurse practitioner, physician assistant or unknown |
| **Antibiotic Prescribed** | Based on review of the visit notes and the prescriptions available in the EMR, was an antibiotic prescribed |
| **URI supportive medication, not antibiotic** | Medications prescribed to the patient based on visit notes and prescriptions in the EMR to address URI-like symptoms (e.g., cough syrup, steroids, other medications) |
| **Revisit within one week** | Documentation of a call or clinical visit that occurred up to one week after the initial clinic visit for URI, URI symptoms or antibiotic or other symptomatic medication adverse events |
| **Smoking status** | Documentation in chart that patient was actively smoking, including cigarettes, cigars, or illicit substances, during 1 year prior to the visit |
| **Baseline kidney function** | Estimated Glomerular Filtration Rate (eGFR) in mg/dL, rounded to the nearest whole number, ordered on the day of the visit or obtained within the prior 6 months |
| **Congestive Heart Failure** | Defined by whether there is an existing problem documented in the EMR problem list, a scanned echocardiogram that documents an EF<40%, or documentation of diagnosis by a cardiologist in a cardiology or primary care note |
| **Chronic Pulmonary Disease** | Defined by a diagnosis of chronic obstructive pulmonary disease, pulmonary fibrosis, bronchiectasis, or lung transplant on the EMR problem list, pulmonary function tests indicating moderate or severe obstruction or restriction, or a documented long-term pulmonary disease by a pulmonologist in a pulmonology or primary care note |
| **Immunosuppression** | Actively receiving chemotherapy or on medications that suppress the immune system including prednisone (long-term use), mycophenolate, leflunomide, tacrolimus, sirolimus, cyclosporine, rapamycin, azathioprine, etanercept, infliximab, adalimumab, abatacept, rituximab, hydroxychloroquine |
| **Insurance Status** | Insurance documented at the time of the visit in the EMR: Medicare, Medicaid, Private, Tricare or Not Listed. Note: Veterans may or may not have some healthcare costs covered by the VA based on their military service. Remaining bills are sent to the Veteran’s insurance company, if applicable. |

**Supplemental Table 3:** **Responder and Nonresponder Veteran and Visit Characteristics**

| Veteran and Visit Characteristic | Responded  N=432 (%) | Did Not Respond  N=844 (%) | Difference  (95% Confidence Intervals) | P Value |
| --- | --- | --- | --- | --- |
| Received Antibiotic | 228 (52.8%) | 434 (51.4%) | -1.4%  (-7.2%, 4.4%) | 0.647 |
| Female | 62 (14.4%) | 160 (19.0%) | 4.6%  (0.4%, 8.8%) | 0.033 |
| Age (years)^a^ | 64.0 (±11.9) | 52.0 (±15.9) | -12.0  (-13.6, -10.4)) | <0.001 |
| Received non-antibiotic prescription for URI symptoms | 232 (53.7%) | 440 (52.1%) | -1.6%  (-7.4%, 4.2%) | 0.595 |
| Called or seen within 1 week of URI visit | 34 (7.9%) | 41 (4.9%) | -3.0%  (-5.9%, -0.1%) | 0.044 |
| Smoked ^b^ | 98 (22.7%) | 320 (37.9%) | 15.2%  (10.1%, 20.4%) | <0.001 |
| Underlying Pulmonary Disease ^c^ | 26 (6.0%) | 39 (4.6%) | -1.4%  (-4.1%, 1.3%) | 0.303 |
| Underlying Congestive Heart Failure | 35 (8.1%) | 34 (4.0%) | -4.1%  (-7.0%, -1.2%) | 0.006 |
| Estimated Glomerular Filtration Rate (mg/dL)^a^ | 77.5 (±21.8) | 84.6 (±22.8) | 7.0  (4.2, 9.8) | <0.001 |
| Immunosuppressed | 6 (1.4%%) | 11 (1.3%%) | -0.1%  (-1.4, 1.3) | 0.901 |
| Insurance documented | 340 (78.7%%) | 455 (53.9%) | -24.8%  (-29.9%, -19.7%) | <0.001 |

Mean differences and 95% confidence intervals calculated using two-sample t-test for continuous and binomial data.

^a^ Variables are reported as mean ± standard deviation in units specified

^b^ Cigarettes, cigars, or illicit substances smoked within 1 year prior to visit

^c^ Excludes obstructive sleep apnea and asthma
